# Clinical evidence of Xinbao Pills efficacy on chronic heart failure patients: A protocol for systematic review and meta-analysis

**DOI:** 10.1097/MD.0000000000030764

**Published:** 2022-09-30

**Authors:** Manhua Huang, Zunjaing Li, Ye Fan, Dongli Li, Quanle Liu, Baijian Chen, Zhe Peng, Banghan Ding

**Affiliations:** a The Second Clinical College of Guangzhou University of Chinese Medicine, Guangzhou, Guangdong, China; b Guangdong Provincial Hospital of Chinese Medicine, Guangzhou, Guangdong, China.

**Keywords:** chronic heart failure, meta-analysis, protocol, systematic review, Xinbao Pills

## Abstract

Chronic heart failure (CHF) is a common cardiovascular disease and results in high rate of morbidity, mortality, hospitalizations, and disability, causing medical concern around the world. Xinbao Pill (XBP) has been widely applied to clinical practice for patients with CHF but lacks studies to verify its efficacy. This study provides a protocol of systematic review, with which we will verify the adjunctive efficacy and safety of XBP on CHF with evidence-based studies.

**Methods:**

Included studies will be retrieved according to inclusion and exclusion criteria from five English databases (the MEDLINE via PubMed, the Cochrane Library, EMBASE, the Web of Science and Ovid database), and four Chinese databases (China Science and Technology Journal Database [VIP], Chinese Biomedical Literature Database [CBM], Wan-fang Database, China National Knowledge Infrastructure [CNKI]) from October 1990 to October 2018. The New York Heart Association (NYHA), heart rate and mortality will be marked as major outcomes. We will use RevMan V.5.3 software to calculate the data synthesis and will conduct meta-analysis based on the collected data.

**Results:**

Mortality, NYHA function classification, heart rate, the left ventricular ejection fractions (LVEF), 6-minute walk test (6MWT), hospitalization or rehospitalization, NT-proBNP, and adverse effects will be measured and comprehensively assessed to evaluate the adjunctive effect of XBP on CHF from this systematic review and meta-analysis with current clinical evidence.

**Conclusion:**

The systematic review and meta-analysis will assess the adjunctive effect of XBP in the treatment of CHF with up-to-date clinical evidence.

## 1. Introduction

Chronic heart failure (CHF) is a serious clinical syndrome with impaired ventricular filling or dysfunction due to abnormal cardiac structure or function,^[1,2]^ which has highly prevalent in adult individuals and results in frequent morbidity, mortality, hospitalizations, and disability, especially for older patients.^[3,4]^ As the quality of life of patients with CHF is significantly impacted, patients with CHF have symptoms as severe and distressing as those of cancer patients,^[5,6]^ with a 5-year survival rate of CHF similar to that of malignant tumors.^[7]^ It is reported that the prevalence rate of CHF increases significantly with age, doubling every 10 years since the age of 45, and its 7-year mortality rate is as high as 75%.^[1,8,9]^

Up to date, the treatment of CHF is still based on palliative care with conventional western drugs, but the therapeutic effect is not optimistic for that patients with CHF often have a high rate of hospitalizations and readmissions after systematic and standardized treatment,^[3,8]^ indicating insufficient effect of these present drugs, which may be due to the frequent incidence of complex complications of the CHF and adverse consequences of the drugs.^[10,11]^ Thus they may be limited in use after a long-term use because of the side effects or aggravation of heart failure, highly effective drugs are still under investigation.^[12]^ Adjuvant or substitute drugs are in urgent need for providing adjuvant treatment, reducing the occurrence of side effects and delaying the progression of the CHF. Recently, many studies found proprietary Chinese medicine played an important role in improving the quality of life care of patients with heart disease like CHF and improving their cardiac function or clinical symptoms.^[13,14]^

Xinbao Pill (XBP) is a traditional Chinese medicine compound preparation developed by the Guangdong Institute of Materia Medica. The main ingredients have 9 medicinal materials including datura metelL, cornu cervi pantotrichum, ginseng, aconitum carmichaeli debx, cinnamomum cassia presl, notoginseng radix et rhizoma, moschus berezovskii flerov, cinobufagin venom toad, and dryobalanopsaromaticaGwaertn.f. Many clinical researches showed that XBP had an obvious effect on improving cardiac function and clinical symptoms in patients with CHF safely and effect.^[15,16]^ However, it still lacks high-quality systematic review and meta-analysis to demonstrate the efficacy of XBP on patients with CHF. This present study is performed to verify the adjunctive efficacy and safety of XBP for CHF with clinical evidence-based studies, and systematically elaborate the specific role of XBP for CHF, which will be served as the guidance for a multi-center random control trial in our further research.

## 2. Method

### 2.1. Inclusion criteria for study selection according to PICOs criteria

#### 2.1.1. Participants.

Adult patients ≥18 years old with clinically diagnosis of CHF in accordance with the Guidelines on the Diagnosis and Treatment of Heart Failure will be included. All gender or race will be included. Any patient with cardiac surgery, other serious complications or cardiac assistive equipment like implantable cardioverter defibrillator (ICD) will be excluded. Patients with CHF caused by non-cardiovascular diseases will be excluded as well.

#### 2.1.2. Interventions and comparison.

In eligible researches, the control group was treated with different types of conventional western medicine (e.g., angiotensin-converting enzyme inhibitors [ACEI], b-blocker, aldosterone inhibitor etc), and the intervention of the experimental group regarded XBW for the treatment of CHF base on the conventional western medicine.

#### 2.1.3. Outcomes measures.

The major outcomes including mortality, clinical total effective rate (according to New York Heart Association [NYHA] function classification), and heart rate will be collected from the eligible randomized controlled trials (RCTs). Besides, the left ventricular ejection fractions (LVEF), 6-minute walk test (6MWT), hospitalization or rehospitalization, NT-proBNP, and adverse effects will be also measured as the secondary outcomes to evaluate the adjunctive effect of XBP.

#### 2.1.4. Study design.

Only RCTs of XBP therapy for CHF will be included. Other types of researches like observational studies will be excluded. No language, time, geographical area, sample size or publication status restriction.

### 2.2. Data search method

Five English databases (The MEDLINE via PubMed, the Cochrane Library, EMBASE, the Web of Science, and Ovid database) and 4 Chinese databases (China Science and Technology Journal Database [VIP], Chinese Biomedical Literature Database [CBM], Wan-fang Database, China National Knowledge Infrastructure [CNKI]) will be searched from October 1990 to October 2018. Standards-compliant conference articles and Doctoral or Master theses were also included. Table 1 lists the keywords and MeSH terms used in the search strategy. The search topics will be composed of P + I + C + O + S, P + I + C + O, and P + I + O in order to ensure the highest search rate of literature search. Some non-published paper or detailed data will be obtained by emailing corresponding authors.

**Table 1:** Keywords and MeSH terms in the search strategy.

PICOS	Keywords and MeSH terms
***P***	Chronic heart failure; CHF; heart failure; HF; chronic cardiac failure; cardiac failure; left ventricular dysfunction; myocardia failure; heart dysfunction; cardiac dysfunction;
***I***	Xinbao Pill; XBP; alternative medicine; Chinese medicine; traditional Chinese medicine; traditional medicine; translational medicine;
***C***	Placebo treatment; treatment as usual; conventional medicine; western medicine or with no treatment;
***O***	Major outcomes: mortality, clinical total effective rate (according to NYHA function classification) and heart rate;Secondary outcomes: LVEF; 6MWT; hospitalization or rehospitalization; NT-proBNP; adverse effects;
***S***	Random control trials; RCTs; random sampling;

CHF = chronic heart failure, HF = heart failure, LVEF = left ventricular ejection fraction, NT-proBNP = N-terminal pro-B-type natriuretic peptide, NYHA = New York Heart Association, 6MWT = 6-minute walk test, RCTs = randomized controlled trials, XBP = Xinbao Pills.

### 2.3. Study selection

**Figure 1.**
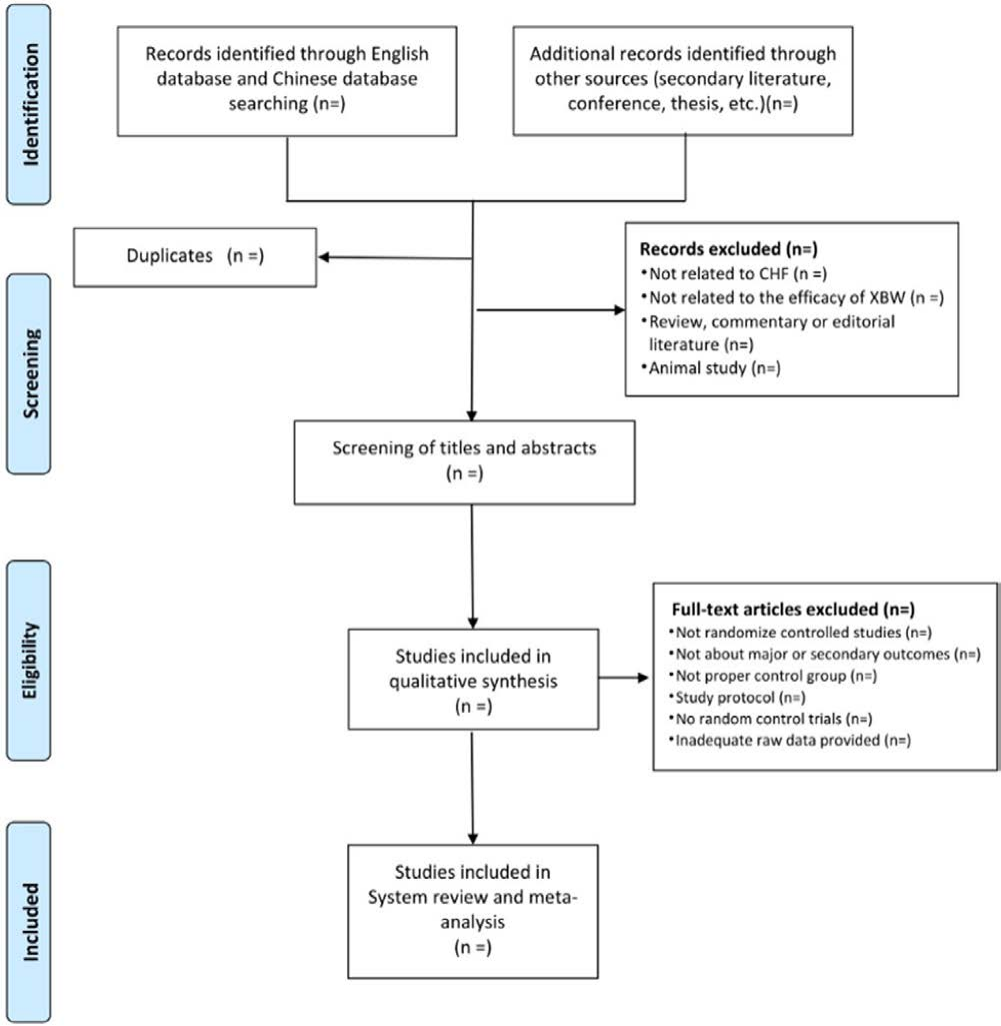
PRISMA flow chart of study selection process in the systematic review. PRISMA = Preferred Reporting Items for Systematic Review and Meta-analysis.

All the random control studies will be screened by two independent researchers according to titles and abstracts firstly before full texts are further evaluated. Duplicate literature will be removed. Studies removed after full text review will be recorded with specific exclusion reason. All the RCTs will be compared with control groups in the way of all types of conventional western drugs treatment. Any selected study will fulfill the requirements of inclusion criteria. Two experienced authors translated the full Chinese reports, and his translations will be clarified by a third author for further evaluation. Disagreements will be resolved by discussion for consensus among the reviewers. The selection process of eligible papers is shown in a Preferred Reporting Items for Systematic Review and Meta-analysis (PRISMA) flow diagram (Fig. 1).^[17,18]^

### 2.4. Risk of bias assessment

The review will assess bias of the studies with the Cochrane Risk of Bias tool consisting of following items (random sequence generation and allocation concealment [selection bias]; binding of participants and personnel [performance bias]; binding of outcome assessment [detection bias]; incomplete outcome data [attrition bias]; selective reporting [reporting bias]; and other potential sources of bias) and evaluated their methodological quality using the Collaboration Review Manager software (Version 5.3 USA). All the items will be evaluated by 2 independent reviewers, and any divergence will be resolved by discussion or the third reviewer as well.

### 2.5. Data extraction and management

#### 2.5.1. Data extraction.

The same 2 reviewers will extract data independently using the special forms base on all the identified studies. Data extraction will include study characteristics, population characteristics, average age, sample size, duration, details of the treatment and control group, details of the outcomes, and adverse events. All the data and disagreement between the 2 reviewers will be evaluated by a third reviewer who served as an arbiter for a final decision throughout the entire procedure.

#### 2.5.2. Dealing with missing data.

Any insufficiency of the above-mentioned data will be supplemented by contacting the corresponding authors using e-mail or telephone. Potential impact of unavailable data will be added in the discussion part for further evaluation on the results.

#### 2.5.3. Quantitative synthesis.

For continuous data, when the change of the mean ($Mean_{Change}$) and standard deviation ($SD_{Change}$) of inflammation levels in pretreatment and post-treatment were provided, we compared the change from baseline to endpoint; when the mean difference ($Mean_{Change}$ ) were provided without providing mean difference standard error ($SD_{Change}$), we calculate the $SD_{Change}$ with the follow formula. If there are three groups, we will compare the data of conventional therapy group with the data of the XBP plus conventional therapy group. Only the last available time point reported on the outcome if the study has different time points throughout the intervention.


$$\begin{array}{l} 1.Corr=\frac{SD_{baseling}^{2}+SD_{final}^{2}-SD_{change}^{2}}{2\times SD_{baseline}\times SD_{final}} \end{array}$$

$$\begin{array}{l} 2.\;SD_{Change}=\sqrt{SD_{baseline}^{2}+SD_{final}^{2}-(2\times Corr\times SD_{baseline}\times SD_{final}} \end{array}$$

#### 2.5.4. Selection of effects model.

The standard mean difference with 95% confidence interval (CI) will be used to evaluate the continuous data, while dichotomous outcomes will be measured with the rate ratio (RR) with 95% CIs. If $I^{2}$ < 50, we will perform the fixed-effects model to calculate the RR and mean difference, but we will choose the random-effects model if $I^{2}$ ≥ 50.

### 2.6. Analysis of the data

#### 2.6.1. Assessment of heterogeneity.

The chi-squared test will be applied to evaluate the heterogeneity with the cutoff value of $I^{2}$ = 50 according to the guideline of Cochrane Handbook. If $I^{2}$ > 50%, trials will be considered with significant heterogeneity, and subgroup analysis will be necessarily performed to assess the potential heterogeneity sources.

#### 2.6.2. Subgroup analysis.

If it is considered with high heterogeneity, subgroup analysis will be performed to reduce the heterogeneity and ensure the accuracy of results including onset time and duration of illness, age, and number of samples etc to investigate which variables best predict the enhancement effect of Xinbao Pills.

#### 2.6.3. Sensitivity analysis.

Sensitivity analysis will be also applied to evaluate the robustness and reliability of the combined results of included studies. Methodological quality, heterogeneity, studies quality (according to the 4 levers: high, moderate, low, or very low), and sample characteristic will be considered.

#### 2.6.4. Assessment of reporting biases.

We will conduct analysis of Egger publication bias plot and Begg funnel plot with pseudo 95% confidence limits to determine the publication bias in all the literature with sufficient studies (more than 10 trials).

#### 2.6.5. Test sequential experiment.

In order to illustrate and confirm the credibility of our results, we performed a sample size analysis by trial sequential analysis (TSA), which aims to rule out the possibility of false positives.

### 2.7. Ethics approval

All the data will be extracted from the published studies through database without directly relate to patients data, thus not ethical approval is required. The findings of this systematic review will provide implication of the enhancement effectiveness of XBW for CHF. The systematic review will be disseminated in a peer-reviewed journal and published at conference presentations, which will be served as the guidance for a multi-center random control trial in our further research.

## 3. Discussion

Chronic heart failure is a common cardiovascular disease with high risk and incidence increasing as ages.^[7,9]^ In the United States, over 5,000,000 Americans have heart failure with over 800,000 new cases diagnosed annually.^[19,20]^ In Chinese patients with CHF, the prevalence of complications like depressive symptoms and dyspnea is relatedly high,^[21]^ it is significant to improve the clinical symptoms and prevent the occurrence of complications in order to reduce its mortality, hospitalizations, and disability. Long-term use of these drugs including β-blockers, ACEI, and angiotensin II receptor blockers (ARBs) etc can results in side effects for patients,^[22–24]^ and proprietary Chinese medicine could prevent or improve adverse clinical symptoms, enhancing the quality of life of patients with CHF and reducing their distressing feels.

XBW may be an adjunctive and effective treatment for CHF with fewer side effects and adverse events, but the adjuvant efficacy of XBW in the treatment of CHF is uncertain. Therefore, it is necessary to conduct high-quality systematic evaluation and meta-analysis on it. Our standardized methods will provide an objective evidence-based review for XBW in the treatment of CHF. We will use high-quality clinical evidence to demonstrate whether XBW has the effect of improving the clinical symptoms and reducing the occurrence of adverse events on the basis of conventional treatment. In addition, whether XBW combined with conventional treatment can be more effectively to improve the primary and secondary outcomes will be verified. However, there are limitations in this systematic review that may affect the drawn conclusion. First, the included trials are mainly restricted to the published results, which may run risk of publishing bias. Second, different times of onset and types of CHF may lead to heterogeneity in our results in terms of that subgroup analysis may be unavailable if the quantities of included studies are not enough. Thus, in order to avoid these, we will conduct this analysis with more studies and more extra unpublished results as we can as possible.

## Author contributions

Banghan Ding designed the study and made all the decisions when came across disagreement during the analysis. Zunjiang Li and Manhua Huang were respossible for data search, extraction and writing. Manhua Huang, Ye Fan and Dongli Li performed data analysis and evaluated the accuracy of the whole process. Quanle Liu, Baijian Chen and Zhe Peng provided support for modifications of English writing. All authors contributed to the evaluation of review and analysis and approved the final version submitted for publication.

**Conceptualization:** Manhua Huang, Banghan Ding.

**Data curation:** Manhua Huang, Zunjaing Li, Ye Fan, Quanle Liu, Banghan Ding.

**Formal analysis:** Manhua Huang, Zunjaing Li, Ye Fan, Quanle Liu, Zhe Peng.

**Funding acquisition:** Banghan Ding.

**Investigation:** Manhua Huang, Dongli Li.

**Methodology:** Manhua Huang, Zunjaing Li, Ye Fan, Dongli Li, Zhe Peng.

**Software:** Ye Fan, Dongli Li, Quanle Liu, Zhe Peng.

**Supervision:** Banghan Ding.

**Validation:** Zunjaing Li, Banghan Ding.

**Visualization:** Banghan Ding.

**Writing – original draft:** Zunjaing Li, Ye Fan.

**Writing – review & editing:** Ye Fan, Baijian Chen, Banghan Ding.
